# Detection of a *cfr(B)* Variant in German *Enterococcus faecium* Clinical Isolates and the Impact on Linezolid Resistance in *Enterococcus spp*.

**DOI:** 10.1371/journal.pone.0167042

**Published:** 2016-11-28

**Authors:** Jennifer K. Bender, Carola Fleige, Ingo Klare, Stefan Fiedler, Alexander Mischnik, Nico T. Mutters, Kate E. Dingle, Guido Werner

**Affiliations:** 1 National Reference Centre for Staphylococci and Enterococci, Division of Nosocomial Pathogens and Antibiotic Resistances, Department of Infectious Diseases, Robert Koch Institute, Wernigerode Branch, Wernigerode, Germany; 2 Division of Infectious Diseases, Department of Medicine, Medical Center - University of Freiburg, Faculty of Medicine, University of Freiburg, Freiburg, Germany; 3 Department of Infectious Diseases, Heidelberg University Hospital, Heidelberg, Germany, and German Centre for Infection Research DZIF, Heidelberg, Germany; 4 Nuffield Department of Clinical Medicine, Oxford University, John Radcliffe Hospital, Oxford, Oxfordshire, United Kingdom; 5 National Institute for Health Research, Oxford Biomedical Research Centre, John Radcliffe Hospital, Oxford, Oxfordshire, United Kingdom; Universitatsklinikum Hamburg-Eppendorf, GERMANY

## Abstract

The National Reference Centre for Staphylococci and Enterococci in Germany has received an increasing number of clinical linezolid-resistant *E*. *faecium* isolates in recent years. Five isolates harbored a *cfr(B)* variant gene locus the product of which is capable of conferring linezolid resistance. The *cfr(B)*-like methyltransferase gene was also detected in *Clostridium difficile*. Antimicrobial susceptibility was determined for *cfr(B)*-positive and linezolid-resistant *E*. *faecium* isolates and two isogenic *C*. *difficile* strains. All strains were subjected to whole genome sequencing and analyzed with respect to mutations in the 23S rDNA, *rplC*, *rplD* and *rplV* genes and integration sites of the *cfr(B)* variant locus. To evaluate methyltransferase function, the *cfr(B)* variant of *Enterococcus* and *Clostridium* was expressed in both *E*. *coli* and *Enterococcus* spp. Ribosomal target site mutations were detected in *E*. *faecium* strains but absent in clostridia. Sequencing revealed 99.9% identity between *cfr(B)* of *Enterococcus* and *cfr* of *Clostridium*. The methyltransferase gene is encoded by transposon Tn*6218* which was present in *C*. *difficile* Ox3196, truncated in some *E*. *faecium* and absent in *C*. *difficile* Ox3206. The latter finding explains the lack of linezolid and chloramphenicol resistance in *C*. *difficile* Ox3206 and demonstrates for the first time a direct correlation of elevated linezolid MICs in *C*. *difficile* upon *cfr* acquisition. Tn*6218* insertion sites revealed novel target loci for integration, both within the bacterial chromosome and as an integral part of plasmids. Importantly, the very first plasmid-association of a *cfr(B)* variant was observed. Although we failed to measure *cfr(B)*-mediated resistance in transformed laboratory strains the occurrence of the multidrug resistance gene *cfr* on putatively highly mobile and/or extrachromosomal DNA in clinical isolates is worrisome with respect to dissemination of antibiotic resistances.

## Introduction

The oxazolidinone linezolid represents an antibiotic of last resort used to treat severe infections that are caused by multidrug-resistant Gram-positive bacteria such as methicillin-resistant *Staphylococcus aureus* (MRSA) or vancomycin-resistant *Enterococcus* spp. (VRE). According to a recent Threats Report from the Centre for Disease Control and Prevention, MRSA and VRE are listed under serious threats which require public health monitoring (http://www.cdc.gov/drugresistance/threat-report-2013/). Considered as an important nosocomial pathogen VRE can cause life-threatening diseases including septicemia, endocarditis or urinary tract infections. After the approval of linezolid in 2000, emergence of resistant strains upon linezolid therapy has been reported for both VRE and vancomycin-susceptible enterococci (VSE) [1, 2]. Although linezolid-resistant enterococci (LRE) are generally detected at low prevalence in Europe, the National Reference Centre (NRC) for Staphylococci and Enterococci at the Robert Koch Institute recognized an increasing number of LRE in German hospitals [3].

Linezolid targets the ribosomal machinery by inhibiting formation of the translation initiation complex, hence protein synthesis [4]. In addition to mechanisms such as mutations in the 23S rDNA or ribosomal protein genes *rplC*, *rplD* and *rplV*, resistance to linezolid is mediated by acquisition of the *cfr* RNA methyltransferase [5–8]. However, *cfr* not only confers insusceptibility to oxazolidinones, but also to a broad range of antibiotics collectively known as PhLOPS_A_ (Phenicols, Lincosamides, Oxazolidinones, Pleuromutilins and Streptogramin A) [5].

Unlike staphylococci, where a combination of site-specific mutations within the rDNA and/or ribosomal proteins and the expression of *cfr* are jointly responsible for the development of a resistance phenotype [9, 10], reports concerning *cfr*-mediated resistance in enterococci remain scarce. Up to now, the majority of studies described the accumulation of mutations affecting the ribosomal machinery [2, 11, 12]. Although the presence and expression of *cfr* in enterococci has been reported previously, the impact of conferring resistance to enterococci isolates is still under debate [13–17]. It must be noted that two isoforms of the *cfr* gene exist in enterococci, one of which is highly similar to *cfr* from staphylococci and the other one being almost identical to a *cfr*-like gene from *Clostridium difficile* [14, 16, 18]. The latter is now termed *cfr(B)* and located on the composite transposon Tn*6218* [16]. Adding to the complexity of linezolid resistance, enterococci from China and Italy were shown to carry *optrA*, a gene encoding an ABC-transporter capable of mediating oxazolidinone and phenicol insusceptibility [17, 19, 20].

It has been reported that *C*. *difficile* isolates with elevated MICs harbor *cfr* [21]; however, it has been shown just recently that episomal expression of the very same gene confers the PhLOPS_A_ phenotype in *E*. *coli* [22]. Nevertheless, until today it remains to be determined whether *cfr* as a sole mechanism is able to mediate PhLOPS_A_ phenotypic resistances in wildtype *Clostridium*.

In the present study, we analyzed the first clinical *cfr(B)*- positive *E*. *faecium* isolates from German hospital patients. The methyltransferase gene was shown to be a close homolog of *cfr(B)* from American *E*. *faecium* isolates and from *C*. *difficile*. As part of a full length or truncated version of transposon Tn*6218* the *cfr(B)* variant described herein showed novel sites for integration and, most importantly, plasmid-association. Expression of this *cfr(B)* variant from enterococci and of *C*. *difficile* failed to recover the PhLOPS_A_ phenotype in *E*. *coli* and *Enterococcus* spp. However, analysis of a closely related strain set of *C*. *difficile* clinical isolates demonstrates for the first time that the Cfr-like methyltransferase is able to mediate oxazolidinone and phenicol resistance in the important gut commensal and nosocomial pathogen.

## Materials and Methods

### Strains used in this study

Five *E*. *faecium* strains isolated from German hospital patients and exhibiting linezolid resistance (Table 1) were tested positive for *cfr* by PCR as described previously [3, 23]. Two closely related *C*. *difficile* strains were isolated in Oxford, United Kingdom in 2012 and are part of a small cluster of geographically and temporally linked cases (Kate Dingle, personal communication); however, Ox3206 lacks the mobile element *cfr-*Tn*6218* which is present in Ox3196 (HG002389) [24].

**Table 1:** Strains used in this study.

Strain	ST	Origin	Year of isolation	Material	Resistance phenotype^a^	MIC LZD [mg/L]^b^	*cfr*	*van* genotype	mutations in 23S rDNA	mutations in *rplC*/L3	mutations in *rplD*/L4	mutations in *rplC*/L22	*optrA*^c^
*E*. *faecium*													
UW10882	117	Heidelberg, Baden-Wuerttemberg	2013	abdominal drainage	AMP, CIP, CLI, ERY, GEN, LZD, MFL, PEN, RIF, STR, TET	32	+	-	G2576T~2:4 (WT:M)	WT	WT	WT	-
UW11590	117	Halle, Saxony-Anhalt	2014	rectal swab	AMP, CIP, CLI, ERY, LZD, MFL, PEN, RIF, STR, SXT, TET, TPL, VAN	6	+	*vanA*	-	WT	_211_GGT_213_	WT	-
UW11733	SLV 117	Bielefeld, North Rhine-Westphalia	2014	tracheal secretion	AMP, CIP, LZD, MFL, PEN, RIF, TET, TPL, VAN	8	+	*vanB*	-	WT	WT	WT	-
UW11858	203	Cologne, North Rhine-Westphalia	2014	rectal swab	AMP, CIP, GEN, LZD, MFL, PEN, RIF, STR, TET, TPL, VAN	48	+	*vanA*	-	WT	_211_GGT_213_	WT	-
UW12712	117	Halle, Saxony-Anhalt	2015	rectal swab	AMP, CIP, CLI, ERY, LZD, MFL, PEN, RIF, STR, SXT, TPL, VAN	32	+	*vanA*	G2576T~4:2 (WT:M)	WT	WT	WT	-
*C*. *difficile*													
Ox3196	37	Oxfordshire, UK	2012	unknown	n.d.	6	+	-	-	WT	WT	WT	-
Ox3206	37	Oxfordshire, UK	2012	unknown	n.d.	0.75	-	-	-	WT	WT	WT	-

^a^MIC determined by broth microdilution for *E*. *faecium* only;

^b^MIC determined by Etest^®^;

^c^absence (-) of *optrA* was inferred from whole genome sequencing data; SLV, single locus variant; AMP, ampicillin; CIP, ciprofloxacin; CLI, clindamycin; ERY, erythromycin; GEN, gentamicin; LZD, linezolid; MFL, moxifloxacin; PEN, penicillin; RIF, rifampicin; STR, streptomycin; STX, trimethoprim/ sulfamethoxazole; TET, tetracycline; TPL, teicoplanin, VAN, vancomycin; n.d., not determined; WT, wildtype allele; MT, mutant allele.

### Antimicrobial susceptibility testing

The antimicrobial susceptibility of *Enterococcus* spp. isolates was determined by broth microdilution (determination of minimal inhibitory concentrations, MICs) according to EUCAST and applying EUCAST MIC breakpoints for interpretation of the results (www.eucast.org); Etest^®^ linezolid (LZD) (bioMérieux, Nürtingen, Germany) was used to confirm results. In addition, transformants of *E*. *coli* Top10, *E*. *faecium* 64/3 and *E*. *faecalis* JH2-2 were analysed for susceptibility to LZD, chloramphenicol (CMP) and florfenicol (FFC). Please note that according to the latest version of CLSI document VET01-A4 no clinical breakpoints are provided for FFC. In case of *C*. *difficile* Etest^®^ for LZD and CMP were used to determine the MIC under anaerobic conditions. Please note that there are no MIC clinical breakpoints available for *C*. *difficile*.

### DNA extraction

*Enterococcus* strains were grown overnight in BHI broth at 37°C. *C*. *difficile* were routinely grown at 37°C on Brucella agar supplemented with 5% blood, 1 mg/L vitamin K and 5 mg/L hemin. Air-sealed containers were adjusted to anaerobic conditions using AnaeroGen^™^ sachets (Thermo Fisher Scientific, Germany). DNA was extracted from cell material obtained from agar plates (*Clostridium*) or from overnight cultures (*Enterococcus*) using the DNeasy blood and tissue kit (Qiagen, Hilden, Germany) according to the manufacturer’s instructions.

### Molecular characterization of linezolid resistance and whole genome sequencing

The ratio of 23S rDNA wildtype and mutated alleles was determined as published previously [25]. In brief, amplified PCR products were digested with NheI which discriminates between wildtype and mutated alleles (position G2576T) and fragments were separated thereafter on a microfluidic chip using an Agilent Bioanalyzer 2100 (Agilent Technologies, Santa Clara, CA). Densitometric analysis allows a qualitative assessment of wildtype and mutated alleles as well as quantification of both allele types per isolate. To analyse possible linezolid resistance-mediating mutations *rplC*, *rplD*, *rplV* were examined from whole genome sequence data. To this end, 1 ng of extracted DNA was subjected to library preparation using the Nextera XT Library Prep Kit according to the manufacturers’ instructions (Illumina). Whole genome sequencing (WGS) was performed on a MiSeq benchtop instrument in paired-end with a maximum of 300 bp read length (MiSeq Reagent Kit v3, 600 cycles, Illumina). For enterococci, obtained sequences were compared to genomic data from *E*. *faecium* DO/TX16 (NC_017960), whereas *C*. *difficile* R20291 (FN545816) served as a reference for strains Ox3196 and Ox3206.

### Whole genome comparison of *C*. *difficile* strains

Genomic reads from *C*. *difficile* Ox3196 and Ox3206 were obtained by next generation sequencing (see above) and assembled *de novo* using the open access assembly pipeline a5-miseq [26]. Subsequently, obtained contigs were concatenated and annotated by RAST [27]. Comparison of whole genome content was carried out using the BLAST Ring Image Generator (BRIG) program [28]. Determination of loci which were absent in *C*. *difficile* Ox3206 was performed by mapping of reads to the concatenated and annotated sequence from *C*. *difficile* Ox3196 using the Geneious software (version 9.1.3).

### Phylogenetic analysis of *cfr(B)*-positive *E*. *faecium*

To determine the phylogeny of the 5 *cfr(B)*-positive *E*. *faecium*, Single Nucleotide Polymorphisms (SNPs) were identified by using a pipeline based on BWA v0.7.12-r1039 [BWA-SW; [29]] for mapping to reference strain *E*. *faecium* 64/3 (CP012522) and VarScan v2.3 [30] for variant calling. SNPs located within a 300 bp distance, or less, from each other were excluded as they might represent variants introduced by recent recombination events. A maximum likelihood tree was inferred from a total of 874 SNP positions by utilizing the PhyML algorithm of the seaview program and 1000 iterations for branch validation.

### Determination of Tn*6218-cfr(B)* insertion sites in *E*. *faecium*

To differentiate between plasmid and chromosomal insertion of Tn*6218* in *E*. *faecium* isolates, bacterial genomic DNA was treated with S1 nuclease in order to linearize plasmids which were separated thereafter by PFGE prior to Southern hybridization using a digoxigenin-labelled *cfr*-specific probe (Roche Biochemicals, Mannheim, Germany) [31]. The *cfr*-specific probe was generated using primers as published previously [23] and *E*. *faecium* UW10882 whole genomic DNA as a template.

For further analyses, *de novo* assembly of reads from Illumina WGS was carried out as described above. In order to determine conserved blocks between isolates, obtained *cfr(B)* variant-containing contigs were aligned thereafter using the Geneious software. For elucidation of putative Tn*6218* insertion sites, transposon flanking regions were extracted and BLASTn search was performed. Insertion sites were verified by PCR using oligonucleotides as stated in S1 Table in a PCR reaction with 12.5 μl of 2 x DreamTaq Green PCR Mastermix (Thermo Fisher Scientific, Germany), 0.1 μM of each primer and 0.5 μl of isolated DNA in a total reaction volume of 25 μl. Thermocycler conditions were set to 2 min at 94°C for initial denaturation followed by 30 cycles of 30 sec at 94°C, 30 sec at 55°C (S1 Table) and 1–2 min at 72°C. A final extension was carried out at 72°C for 4 min.

### Cloning of *cfr(B)* variants*-*expressing plasmids

The *cfr(B)* variant gene from *E*. *faecium* UW10882 and *C*. *difficile* Ox3196 was amplified by PCR using Phusion polymerase (Thermo Fisher Scientific, Germany), oligonucleotides cfr_us_BamHI_fw and cfr_ds_SalI_rv3 (S1 Table) and a protocol as follows: 98°C for 30 sec for initial denaturation, 30 cycles of 10 sec at 98°C, 30 sec at 54°C-64.5°C and 60 sec at 72°C, and a final extension for 5 min at 72°C. The primers were designed to bind 220 bp upstream of the start of protein translation and terminate 127 bp downstream of the stop codon. PCR products were digested using BamHI and SalI and ligated to appropriately cut vector pEF-25 before transformation of chemically competent *E*. *coli* Top10. Positive transformants were selected on LB agar plates containing 50 mg/L spectinomycin (SPC) and inserts were verified by Sanger sequencing. Designated plasmids pWJB001 (pEF-25 + *cfr(B)* variant_*Enterococcus*_) and pWJB002 (pEF-25 + *cfr*_*Clostridium*_) were used to electroporate laboratory strains *E*. *faecium* 64/3 and *E*. *faecalis* JH2-2 and transformants were selected on BHI agar supplemented with 200 mg/L SPC [adapted from [32]].

### Expression analyses of the *cfr(B)* variants in *Enterococcus* spp.

Two independent transformants of *E*. *faecium* 64/3 (pWJB001 or pWJB002) and *E*. *faecalis* JH2-2 (pWJB001 or pWJB002) were analyzed with respect to antibiotic insusceptibility. Gene expression of the *cfr(B)* variant targets was evaluated by total RNA extraction and reverse transcription as described elsewhere [33]. Quantification of cDNA was carried out using the DyNAmo ColorFlash SYBR Green qPCR kit (Thermo Fisher Scientific, Germany) in a CFX96 qPCR instrument (Bio-Rad, Germany). Primers cfr_UW10882_qPCR_fw and cfr_UW10882_qPCR_rv (S1 Table) were used at a final concentration of 0.1 μM in a 20 μl reaction containing 5 μl of 1:10 diluted cDNA for target amplification or 5 μl 1:1000 diluted cDNA for amplifying the 16S reference gene [33]. Thermocycler conditions were set to: 2 min at 95°C, 45 cycles of 10 sec at 95°C and 30 sec at 60°C and a melting curve was determined in the range from 60–98°C.

### Filter-mating experiments

Transfer capabilities of transposon Tn*6218* was determined by filter-mating experiments using *E*. *faecium* 64/3, *E*. *faecalis* OG1RF or *E*. *faecalis* JH2-2RF as recipient strains and as published previously [34]. The following isolates were used as donors: *E*. *faecium* strains UW10882, UW11590, UW11733, UW11858, UW12727 and *C*. *difficile* Ox3196. The bacterial mixture was placed on a nitrocellulose filter on BHI agar plates and in case of *Clostridium* under anaerobic conditions to allow overnight transfer of *cfr* between the two species. Putative transconjugants of filter-mating experiments were then selected on BHI agar plates containing i) 10 mg/L FFC, 30 mg/L rifampicin (RIF) and 20 mg/L fusidic acid (FUS); ii) 5 mg/L CMP, 30 mg/L RIF and 20 mg/L FUS or iii) plates containing 30 mg/L RIF and 20 mg/L FUS only. Further, putative transconjugants were screened for acquisition of *cfr* by PCR.

### Nucleotide accession numbers

Raw reads are available from the SRA under accession number SRP078305.

## Results

### Description of bacterial isolates

Five clinical LRE isolates, collected between 2013 and 2015 from German hospital patients, were tested positive in *cfr*-specific PCR screening experiments [3]. The strains exhibited a multidrug-resistant phenotype with varying levels of MICs to linezolid ranging from 6 mg/L to 48 mg/L (Table 1). Three isolates (UW10882, UW11590, UW12712) were of sequence type (ST) 117, but originated from two distinct geographical regions (Table 1). Further, 4/5 strains were positive for either *vanA* or *vanB* whereas UW10882 did not harbor a glycopeptide resistance gene (Table 1). Investigation of the *cfr* locus (outlined below) revealed a highly similar genomic structure to a *cfr* gene located on transposon Tn*6218* from *C*. *difficile*. Thus, we further analyzed the *cfr*-positive isolate *C*. *difficile* Ox3196 (ST37) (HG002389) and compared it to *C*. *difficile* Ox3206 (ST37) a close but *cfr*-negative relative of Ox3196. Phenotypic characterization revealed an 8-fold difference in linezolid MIC between the *cfr*-positive and the *cfr*-negative *C*. *difficile* isolate (Table 1). Clostridia were also analyzed for resistance to chloramphenicol, thereby showing MICs of 24 mg/L for Ox3196 compared to 2 mg/L for Ox3206, respectively.

### Analyses of linezolid resistance-associated DNA mutations and of the *cfr(B)* variant methyltransferase gene

Genotypic characteristics likely associated with linezolid resistance were determined for each of the five *Enterococcus* and the two *Clostridium* isolates. First, mutations in the 23S rDNA were analyzed by NheI restriction. Different ratios of wildtype to mutated alleles were found for *E*. *faecium* UW10882 and UW12712 (Table 1). No respective mutation of the 23S rDNA alleles was detected in *C*. *difficile* or the remaining *E*. *faecium*. Second, WGS was carried out to extract genomic information regarding mutations in loci encoding for ribosomal target proteins. This yielded two mutations within *rplD* (L4) for *E*. *faecium* UW11590 and UW11858 (Table 1). A triplet nucleotide insertion (_211_GGT_213_) and thus addition of amino acid glycine at the respective position was detected in both cases. Interestingly, *Enterococcus* isolates harboring 23S rDNA mutations showed no additional alterations in genes encoding for the three ribosomal proteins investigated (Table 1). In case of *C*. *difficile* no differences were detected between the two isolates. However, synonymous mutations in *rplC* were found when compared to the reference genome *C*. *difficile* R20291 (not shown). Moreover, the entire set of WGS derived reads was mapped to the recently identified oxzolidinone and phenicol resistance gene *optrA* [19]; however, none of the strains carried *optrA* (Table 1). In general, no apparent correlation was observed between the occurrence of certain nucleotide mutations and the level of linezolid resistance.

The *cfr* genes from the investigated *E*. *faecium* and *C*. *difficile* isolates were extracted from WGS data and the predicted protein sequences were aligned thereafter to previously published Cfr from enterococci and staphylococci [16, 35]. Notably, Cfr from *E*. *faecium* UW12712 showed 99.7% identity to the recently identified Cfr(B) from *E*. *faecium* of human origin [16], hence differing in a single amino acid at position 314 (K314E) only. Cfr of the remaining *Enterococcus* exhibited 99.4% identity to Cfr(B) due to an additional mutation at position 247 which results in an arginine by lysine substitution (R247K). On the basis of homology, Cfr(B)-like sequences of the five *E*. *faecium* isolates were consequently termed Cfr(B) variants.

The Cfr(B) variants were 100% identical between *E*. *faecium* UW10882, UW11590 and UW11733. Except for Cfr(B) from *E*. *faecium* UW12712, Cfr(B) variants from the clinical *E*. *faecium* isolates showed 99.7% identity to Cfr from *C*. *difficile* with a single isoleucine to valine (I294V) substitution. Likewise, *E*. *faecium* UW12712 displayed this very same mutation, but additionally carried the already mentioned lysine by arginine substitution at position 247 (K247R) compared to Cfr from *C*. *difficile*. Similar to Cfr(B), Cfr(B) variants of the here described *E*. *faecium* strains were remarkably different to Cfr from staphylococci (74.1% for *E*. *faecium* UW12712 and 78% for the other four *E*. *faecium* isolates).

### Cfr-dependent linezolid resistance in *C*. *difficile*

**Fig 1:**
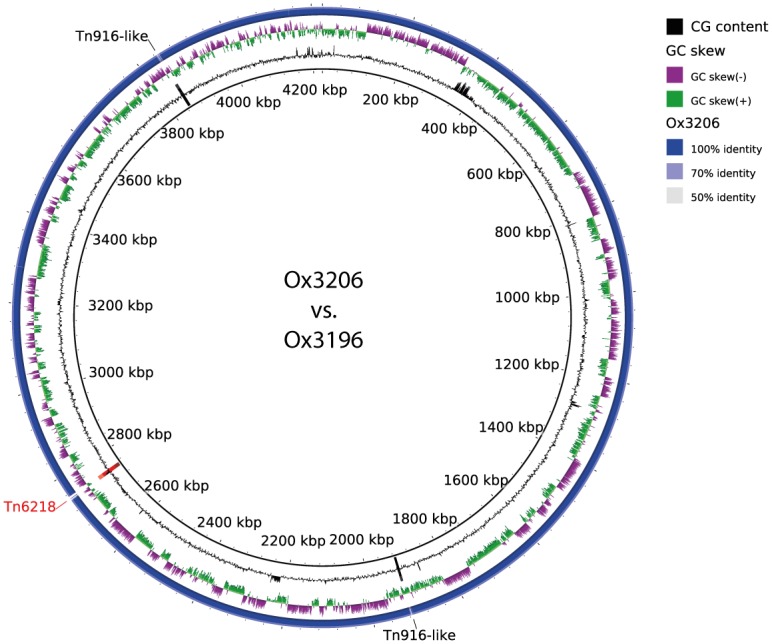
Whole genome comparison of *C*. *difficile* Ox3196 and Ox3206. BRIG alignment of the concatenated genomic contigs obtained by WGS revealed two regions which are absent in *C*. *difficile* Ox3206 and harbor Tn*916*-like related genes. Likewise, the entire transposon Tn*6218* (red), containing the *cfr* resistance locus, is present in *C*. *difficile* Ox3196 only.

NheI restriction analysis and WGS comparisons of *C*. *difficile* Ox3196 (*cfr*-Tn*6218*-positive) and *C*. *difficile* Ox3206 (*cfr*-Tn*6218*-negative) revealed no difference in linezolid-associated genomic mutations (Table 1). It has recently been shown that ectopic expression of *cfr* from *C*. *difficile* in *E*. *coli* confers resistance to the recipient bacteria [22]; however, *cfr*-dependent insusceptibility to linezolid in *C*. *difficile* is still under debate [21, 22]. In order to verify *cfr*-mediated linezolid and chloramphenicol resistance in *C*. *difficile* (see above) and to exclude the lack of genomic content other than Tn*6218* in strain Ox3206 as the source for reduced susceptibility (Table 1) whole genome comparison of both isolates was carried out utilizing the BLAST Ring Image Generator (BRIG) program. As illustrated in Fig 1 only Tn*6218* and two Tn*916*-like elements were absent in *C*. *difficile* Ox3206 (Fig 1).

Subsequent RAST annotation and detailed examination of the genomic regions encoded by the two Tn*916*-like elements produced open reading frames for a Tn*916* transcriptional regulator, a putative transposon integrase (only one Tn*916*-like element), multiple hypothetical protein and two dihydrofolate reductase genes (one Tn*916*-like element only) (not shown). Thus, it is feasible to attribute elevated linezolid MICs of *C*. *difficile* Ox3196 to the presence of the *cfr*-containing transposon Tn*6218*.

### Determination of the genetic environment of the *cfr(B)* variant gene locus of clinical *E*. *faecium* isolates

**Fig 2:**
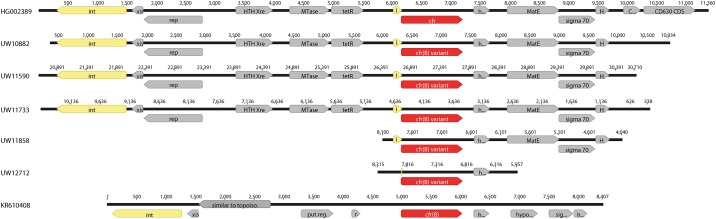
Schematic representation of Tn*6218* in clinical *E*. *faecium* isolates. Different variants of Tn*6218*-like elements were detected in the five German *cfr(B)*-positive *E*. *faecium* clinical isolates (UW numbering). UW12712 exhibits a truncated element encoding solely for the *cfr(B)* variant and a hypothetical protein of the original composite transposon Tn*6218* of *C*. *difficile* (HG002389). *E*. *faecium* UW10882, UW11590 and UW11733 all harbor highly similar or even complete identical (UW11590 and UW11733) Tn*6218*-like structures and gene contents. A comparison of Tn*6218* of the clinical isolates and of *C*. *difficile* Ox3196 to the mobile element of the American *E*. *faecium* isolate 448-18961R (KR610408) revealed an almost entirely different gene composition. Numbers represent the length of the published sequence or obtained contig, respectively. Abbreviations: int, integrase; xis, excisionase; rep, putative topoisomerase; HTH Xre, putative transcriptional regulator; i, integrase core domain fragment; h and hypo, hypothetical protein; sigma 70, sigma 70 region 4; H, HTH motif coding sequence (CDS); C, CD31680 CDS; r, regulatory protein.

It has been reported that *cfr* from *C*. *difficile* and *cfr(B)* from *E*. *faecium* are encoded by transposon Tn*6218* and Tn*6218*-like elements, respectively [16, 24]. In order to disclose the genetic environment of the *cfr(B)* variants from the German clinical *E*. *faecium* isolates, next generation sequencing was carried out and *cfr(B)*-containing contigs were examined after *de novo* assembly in a way that Tn*6218*-like structures were extracted and compared to Tn*6218* from *C*. *difficile* (HG002389) and to *cfr(B)* and adjacent regions from *E*. *faecium* (KR10408) (Fig 2).

**Fig 3:**
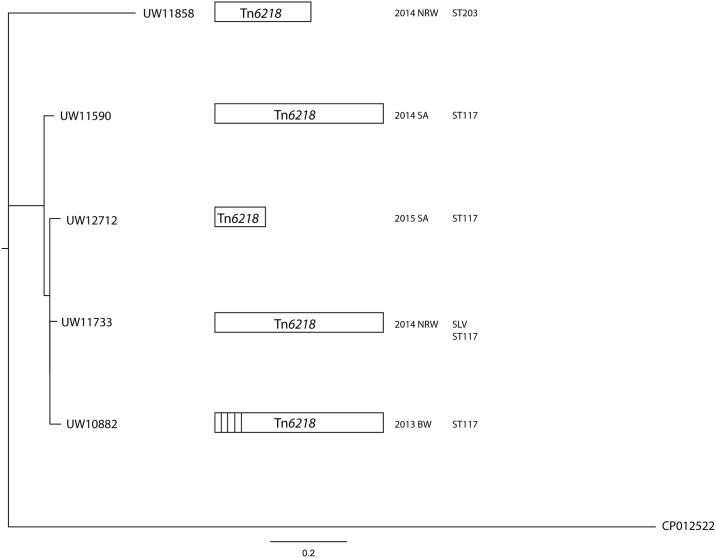
Maximum likelihood analysis of German *cfr(B)*-positive *E*. *faecium*. hylogenetic analysis inferred from mapping of whole genome sequencing reads to reference genome *E*. *faecium* 64/3 (CP012522) did not indicate a close relationship between the German *E*. *faecium* isolates. For comparative reasons, the length of the putative transposon Tn*6218* sequence is indicated by boxes. Strain characteristics such as year and federal state of isolation as well as sequence types are listed. Abbreviations: NRW, North Rhine-Westphalia; SA, Saxony-Anhalt; BW, Baden-Wuerttemberg; SLV, single locus variant.

*E*. *faecium* UW10882, UW11590 and UW11733 harbor a Tn*6218*-like element highly similar to Tn*6218* from *C*. *difficile* (HG002389) (Fig 2). Nucleotide alignment revealed an identical and conserved block between the four strains of 6,900 bp and spanning the methyltransferase locus. However, ORFs located upstream of the *cfr(B)* variant and encoding an integrase, excisionase and putative topoisomerase were less than 97% conserved between *Enterococcus* and *Clostridium* (not shown). Interestingly, *E*. *faecium* UW11590 and UW11733 displayed an identical stretch of 9,733 bp including the integrase locus that differed when compared to UW10882 (schematic representation in Fig 3). In contrast, examination of regions adjacent to the *cfr(B)* variant of *E*. *faecium* UW11858 and UW12712 indicated that those strains only harbor a truncated version of Tn*6218* (Fig 2). The *cfr(B)* variant locus of *E*. *faecium* UW11858 displayed a conserved region of 4,330 bp when compared to Tn*6218* from *C*. *difficile* and UW12712 exhibited even less identity to the respective mobile element (in total 1,788 bp; Fig 2). It is worth noting that the genetic environment of *cfr(B)* variants of *E*. *faecium* isolates described in this study was strikingly different to the *cfr(B)* locus of the American *E*. *faecium* isolate 448-18961R (KR610408) (Fig 2).

### Phylogenetic relationship of German *cfr(B)*-positive *E*. *faecium* isolates

Strain characteristics with respect to sequence type, phenotypic profile, genotype of the vancomycin resistance locus and region of isolation suggests a possible link between isolates UW11590 and UW12712 (Table 1), although the latter only carries a truncated version of the Tn*6218*-like element (Fig 2). Hence, mapping of sequencing reads to reference *E*. *faecium* 64/3 was performed to infer the genetic relationship of the five *E*. *faecium* strains. As a result, 874 SNPs served as the basis for Maximum Likelihood analysis by the PhyML algorithm. All the isolates were only distantly related to each other (Fig 3).

Further, a distance matrix extracted from Neighbor-Joining analyses in Geneious demonstrated that *E*. *faecium* UW11590 and UW12712 were separated by 56 SNPs, thus excluding an immediate close relatedness. In summary, WGS analyses and epidemiological and genotypic data of the strains as well as their resistance profiles (Table 1) did not indicate a direct interrelationship or clonal spread of *cfr(B)*-positive *E*. *faecium* in Germany.

### Elucidation of Tn*6218*-like insertion sites

**Fig 4:**
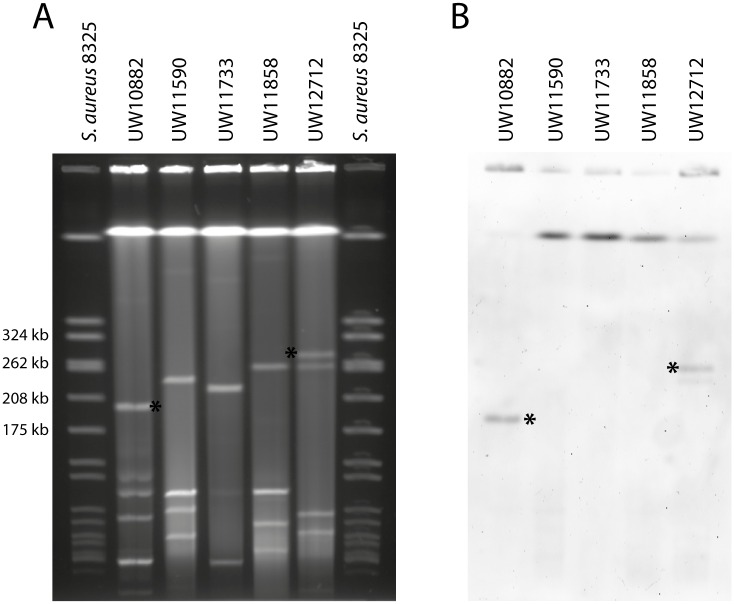
Determination of plasmid content and localization of the *cfr(B)* variant in the five *E*. *faecium* isolates. (A) Separation of whole and linearized plasmid content by S1-PFGE. (B) Hybridization using a *cfr*-specific probe was carried out after transfer of plasmids to a nitrocellulose membrane. The asterisks indicate a *cfr*-positive signal (in B) and the corresponding plasmid (in A). *S*. *aureus* 8325was used as a reference strain for estimation of plasmid sizes.

Tn*6218* of *C*. *difficile* was reported to be integrated into the bacterial chromosome. Hence, Southern hybridization experiments of whole plasmid content after S1-nuclease treatment and PFGE and by using a *cfr*-specific probe was performed for the *cfr(B)* variant-expressing *E*. *faecium* isolates. As depicted in Fig 4, plasmid-association is suggested for *E*. *faecium* UW10882 and UW12712 with an estimated plasmid size of approximately 200 kb and 300 kb, respectively (Fig 4A and 4B).

It is worth mentioning that hybridization experiments of phenol/chloroform extracted whole plasmid content verified results obtained by S1-PFGE (not shown). For detailed disclosure of insertion sites, sequences of regions adjacent to the mobile element were extracted from *cfr(B)*-carrying contigs and BLASTn search was performed to reveal respective target genes or intergenic regions. Table 2 summarizes putative insertion sites which were verified by PCR as indicated.

**Table 2:** Insertion sites of the Tn*6218*-like element in clinical *E*. *faecium* isolates.

Strain	Length of *cfr*-contig after *de novo* assembly	Tn*6218* insertion site/adjacent regions	localization in reference	verified by PCR	localization determined by Southern Blot
*E*. *faecium*					
UW10882	10,934 bp	us: fnr ↨ HMPREF0351_12931ds: HMPREF0351_13032 ↨ HMPREF3051_13033	plasmid pDO3 (CP003586)	yes, both loci	plasmid
UW11590	33,080 bp	EFAU085_RS14120 ↨ EFAU085_RS14125	chromosome (NC_021994)	yes	chromosome
UW11733	25,565 bp	within EFAU085_RS09245	chromosome (NC_021994)	yes	chromosome
UW11858	42,559 bp	within Tn*5386*-like element	chromosome (NC_021994)	n.a.	chromosome
UW12712	38,570 bp	unknown sequence	putatively plasmid (no reference)	yes	plasmid

us, upstream; ds, downstream;

^↨^, insertion site of Tn*6218*;

n.a., not applicable

However, insertion sites were questionable in some cases, as for instance *in silico* analysis of up- and downstream sequences of *E*. *faecium* UW10882 Tn*6218* yielded two different target regions which, according to reference plasmid pDO3 from *E*. *faecium* DO/TX16 (CP003586), are located >95 kbp apart from each other. Nevertheless, both loci showed positive PCR results by amplifying Tn*6218* and adjacent sequences from *E*. *faecium* UW10882 and S1-PFGE results are in support of plasmid localization (Table 2, Fig 4).

Also, we failed to reveal the integration site of the truncated version of Tn*6218* (Fig 2) from *E*. *faecium* UW11858 (Table 2). Adjacent regions yielded no hits with BLASTn or showed similarity to conjugational genes from *C*. *perfringens* pJIR4150 plasmid sequences or, to a lesser extent of identity (78% - 83%), to hypothetical proteins of Tn*5386* from *E*. *faecium* D344R (not shown). This suggests an environment linked to mobile genetic elements of various origins. It remains elusive whether the ancestor of UW11858, eventually having received the *cfr(B)* variant, already harbored a Tn*5386*-like element or related MGEs.

Adding to the complexity, target sites for *cfr(B)*-Tn*6218* insertion in *E*. *faecium* UW12712 remain speculative as BLASTn of the entire 38,570 bp *cfr*-contig revealed homologous regions which could either be allocated to transposon and genomic content or plasmid sequences (not shown). For instance, about half of the contig showed 93% identity to parts of a conjugation module and transposase sequences of *C*. *perfringens* plasmid pJIR4150; however, reconstruction of a putative novel plasmid was not achieved which is most likely due to the size and highly plastic nature of enterococcal extrachromosomal DNA. Nevertheless, plasmid-association as proposed *in silico* is in accordance with results obtained by Southern hybridization which demonstrated plasmid integration of the *cfr(B)* variant locus for UW12712 (Fig 4). Integrity of the contig was verified by PCR bridging the truncated Tn*6218*-like fragment and flanking regions (Table 2).

In summary, Tn*6218*-like elements showed novel sites of integration in German *E*. *faecium* clinical isolates.

### Transformation of *cfr*-carrying plasmids into *E*. *faecium* and *E*. *faecalis* laboratory strains and determination of a *cfr(B)* variant-mediated resistance phenotype

In order to exclude that mutations in linezolid resistance-associated genes (see above) are the sole source for linezolid insusceptibility in *cfr(B)*-positive *E*. *faecium* isolates, the *cfr(B)* variant gene locus of both *E*. *faecium* UW10882 and of *C*. *difficile* Ox3196 was cloned and expressed in *E*. *faecium* 64/3 and *E*. *faecalis* JH2-2, respectively. Acquisition of the *cfr(B)* variant was verified by colony PCR (not shown) and *cfr*-mediated resistance to various antibiotics was determined by broth microdilution (S2 Table). However, introduction of the *cfr(B)*variant-containing vectors did not confer resistance to the transformants or elicited elevated MICs to the antibiotics tested (S2 Table). Quantitative PCR was carried out from reverse transcribed RNA to exclude insufficient transcription as causative for the lack of a resistance phenotype. Thereby, *cfr(B)* variant transcripts were detected at high levels in all strains analyzed (not shown).

### Conjugational transfer of *E*. *faecium* and *C*. *difficile* Tn*6218*

Whole genome sequencing revealed the presence of putatively intact Tn*6218* and Tn*6218*-like structures in *C*. *difficile* Ox3196 and *E*. *faecium* UW10882, UW11590 and UW11733, respectively (Fig 2). Also and according to results from Southern hybridization utilizing a *cfr*-specific probe, *E*. *faecium* UW10882 and UW12712 seem to carry the *cfr(B)* variant on a plasmid (Fig 4). In order to examine transfer capabilities and thus spread of the resistance cassette, filter-mating experiments were carried out using *E*. *faecium* 64/3 and *E*. *faecalis* OG1RF or JH2-2RF as susceptible recipient strains. Conjugation between *C*. *difficile* and enterococci was investigated under anaerobic conditions. The antibiotics chloramphenicol or florfenicol were used as selective markers; however, putative transconjugants were also examined from non-selective plates in case transfer and expression of the *cfr(B)* variant fails to mediate a resistance phenotype as seen in previous experiments (see above). Unfortunately, screening of putative transconjugants by *cfr*-specific PCR yielded no positive candidates after repeated filter-mating experiments and multiple conditions tested.

## Discussion

Determination of resistance development to antibiotics of last resort, such as resistance to the oxazolidinone linezolid, is of global importance with respect to remaining treatment options for severe infections caused by multidrug-resistant bacteria. As for VRE, linezolid therapy represents a valuable alternative since the general prevalence of LRE has remained constant ≤ 1% for the last few years (http://www.ars.rki.de) [36]. We herein describe the detailed investigation of the first *cfr(B)* variant*-*containing *E*. *faecium* strains isolated from German hospital patients [3]. *Cfr* and recently *cfr(B)* have already been shown to be distributed across various bacterial species [16, 22, 37] and outbreaks involving *cfr*-encoding Gram-positive bacteria haven been reported [38]. However, the impact of *cfr* on linezolid insusceptibility in *E*. *faecium* is still questionable, as resistance development is based on multiple mechanisms including mutations affecting the ribosomal machinery [8]. In accordance, detailed molecular investigation of strains from the present study showed the combination of DNA mutations and presence of a *cfr(B)* variant, with isolate UW11733 being the sole exception. No correlation of an increase in linezolid MIC with respect to acquisition of multiple resistance mechanism could be detected. This aspect makes it difficult to attribute linezolid resistance to the presence of the *cfr(B)* variant in the *E*. *faecium* clinical isolates, not least because it has been reported that existence and expression of *cfr* in *E*. *faecalis* does not necessarily mediate a resistance phenotype [15]. In concordance with the data from Liu *et al*., we performed heterologous expression of the *cfr(B)* variant in *E*. *coli* and laboratory strains of *E*. *faecium* and *E*. *faecalis*, but also failed to observe resistance development in the respective transformants. It must be noted that the *cfr* gene from *E*. *faecalis* and the *cfr(B)* variant from German *E*. *faecium* strains are markedly different. Nonetheless, recent examinations of *cfr(B)* from *E*. *faecium* and of *cfr* from *C*. *difficile* clearly demonstrated that expression in either *S*. *aureus* or *E*. *coli* confers a similar resistance phenotype [16, 22]. Hansen and Vester [22] performed a codon optimization in order to express and analyze the *cfr* gene in *E*. *coli*, which might explain the lack of resistance of our *E*. *coli* transformants harboring *cfr* from *C*. *difficile* (not shown). However, this does not explain the missing phenotype when expressing the *cfr(B)* variant of *E*. *faecium* and of *C*. *difficile* Ox3196 in laboratory strains of *E*. *faecium* and *E*. *faecalis*. Interestingly, Deshpande and coworkers reported about isolate *E*. *faecium* 18961 which, although carrying *cfr(B)*, failed to develop the full PhLOPS_A_ phenotype. As for instance, strain 18961 showed only low MIC levels for clindamycin [16]. Likewise, the German isolates UW11733 and UW11858 do not exhibit the entire PhLOPS_A_ resistance pattern and displayed MIC values for clindamycin below the detection limit of ≤ 0.5 mg/L (not shown). Hence, it remains to be determined whether Cfr(B) methyltransferases and variants thereof are causative for resistance development to five classes of antibiotics in enterococci. Nonetheless, the *cfr(B)* variant of UW11733 is highly likely to impact linezolid insusceptibility (MIC 8 mg/L), as the strain does not carry any additional chromosomal mutation nor does it harbor the recently identified oxazolidinone and phenicol resistance gene *optrA* (Table 2) [17, 19].

As mentioned above, Hansen and Vester confirmed that *cfr* from *C*. *difficile* is able to confer resistance to *E*. *coli* transformants [22]; however, they did not pursue to examine *cfr*-mediated resistance in the parental *Clostridium*. Also, a previous study could not clearly link the presence of *cfr* to elevated levels of linezolid MICs in *C*. *difficile* [21]. By analyzing a closely related set of *C*. *difficile* isolates differing only in the presence or absence of *cfr*- encoding Tn*6218* we demonstrated for the first time that upon acquisition of the transposon *C*. *difficile* exhibits elevated MICs to linezolid (Table 1) and chloramphenicol (see above). Importantly, linezolid is not prescribed for the treatment of *C*. *difficile* infections. Nevertheless, clostridia are part of the human gut microbiota which represents a reservoir for active and mobile resistance determinants [39]. Moreover, *C*. *difficile* are known to harbor various conjugative and non-conjugative transposable elements, some of which encode for chloramphenicol or tetracycline resistance determinants [40]. Hence, antimicrobial selective pressure imposes a risk to clinical settings and raised the question whether linezolid resistance encoded by the putatively mobile genetic element (MGE) Tn*6218* is transferable between gut commensal organisms. Successful transfer of MGEs other than Tn*6218* has frequently been demonstrated to occur between *C*. *difficile* and *Enterococcus* spp. [41–43]. Three of the five *E*. *faecium* strains analyzed in this study harbor a Tn*6218*-like transposon highly similar to the one from *C*. *difficile* and thus could potentially disseminate the resistance to receptive bacteria. Also, isolates UW10882 and UW12712 carry the *cfr(B)* variant on plasmids (Fig 4) which were reported to easily facilitate transfer of linezolid resistance [18, 44, 45]. Also, plasmid-encoded *cfr* was shown to transfer frequently between enterococci or staphylococci [15, 18, 37, 45] and in some cases even within bacterial phyla [35]. A series of filter-mating experiments using all five clinical *cfr(B)*-positive *E*. *faecium* isolates and *C*. *difficile* Ox3196 as donors were conducted but failed to transfer the resistance to laboratory strains of *E*. *faecium* or *E*. *faecalis*, respectively. The most obvious explanation is represented by the inability of the *cfr(B)* variant to confer elevated MICs to florfenicol (see above) which was used as a selective marker in filter-mating experiments. Further, Tn*6218* belongs to the family of non-conjugative transposons and thus lacks a functional conjugation machinery. Mobilization of MGEs, though possible [46], would require specific host determinants which might be absent in our donor strains tested In accordance, some studies also report about ineffective transfer from *Enterococcus* spp. to staphylococci or *vice versa* [18, 47]. Thus, the questions of whether or not the *cfr(B)* variant could to be disseminated amongst enterococci by conjugation requires further investigation.

In general, *cfr* is an integral part of multiple MGEs incorporated into plasmids or the chromosomal backbone [37]. The various Tn*6218*-like elements of the German *E*. *faecium* strains revealed target sites for transposon integration which were entirely different to the ones reported for *cfr* and *cfr(B)* from enterococci so far [16, 37]. Phylogenetic investigations did not indicate clonal relatedness of the five *E*. *faecium* isolates and thus suggest an independent acquisition of the Tn*6218*-like and *cfr(B)* variant-containing element. In addition, chromosomal rearrangements preceding or following the uptake of the resistance determinant are highly likely for isolate UW10882, as *cfr*-flanking regions yielded two different insertion sites which, according to reference pDO3, would be located far apart from each other. Last but not least, the two truncated variants of the Tn*6218*-like element investigated herein document the modular structure of such elements [24] and add to the pool of newly composed *cfr*-containing MGEs.

In conclusion, we could demonstrate for the first time that *cfr* is able to mediate linezolid resistance in a clinical *C*. *difficile* isolate. Most importantly, this is the first report of *cfr(B)* in German *E*. *faecium* strains and the first mentioning of plasmid-localization of a *cfr(B)* variant in enterococci of clinical origin. As a consequence, surveillance of *cfr*-positive strains and detailed investigation of the genetic requirements for resistance acquisition and expression is essential to prevent emergence and spread of untreatable multidrug-resistant pathogens.

## Supporting Information

S1 Table Oligonucleotides used in this study. (DOCX)

S2 Table MIC determination of *E*. *faecium* and *E*. *faecalis* transformed with either *cfr(B)* variants from *E*. *faecium* or *C*. *difficile*. (DOCX)
