# Supplementary material for: Detection of a cfr(B) Variant in German Enterococcus faecium Clinical Isolates and the Impact on Linezolid Resistance in Enterococcus spp
Source: PLoS One. 2016 Nov 28;11(11):e0167042. doi: 10.1371/journal.pone.0167042 (PMC5125667; doi:10.1371/journal.pone.0167042)
Supplement: S1 Table — (DOCX) [file pone.0167042.s001.docx]

# S1 Table. Oligonucleotides used in this study.

| **Oligonucleotide** | **Sequence**  **[5’-3’]** | **Annealing**  **temperature**  **[°C]** | **Purpose** |
| --- | --- | --- | --- |
| UW10882_isTn6218_P1_fw | - CAAGCCATTATTCCTTCTTCT - | 55 | vPCR |
| UW10882_isTn6218_P1_rv | - CATTATGCAGGTTGATGAAGAAT - | 55 | vPCR |
| UW10882_isTn_P2_rv2 | - CTGATATAGGGTAAAGATGCTT - | 55 | vPCR |
| UW10882_isTn_P3_rv | - CATTAGCAATATATAAAAGAAGGT - | 55 | vPCR |
| UW10882_isTn_usc_rv | - CATCTTTACCCTATATCAGCGAT - | 55 | vPCR |
| UW11590_isTn6218_P1_fw | - CAGCACGATTATTAAGTGAGT - | 55 | vPCR |
| UW11590_isTn6218_P1_rv | - CATGTATCACGTTCTGCTATT - | 55 | vPCR |
| UW11590_isTn6218_P2_fw | - CGTCTAATCGCATAAGAAGT - | 55 | vPCR |
| UW11590_isTn6218_P2_rv | - CTCGGCATTTGAAGTCGTACT - | 55 | vPCR |
| UW11733_isTn6218_P1_fw | - CGCCTCACAGACTCTATTCT - | 55 | vPCR |
| UW11733_isTn6218_P1_rv | - CGCAGAAGAAGTAACGGATT - | 55 | vPCR |
| UW11733_isTn6218_P2_fw | - CCACATATCACACCGCAT - | 55 | vPCR |
| UW11733_isTn6218_P2_rv | - CAATTGAAGGGATGATGTAAGT - | 55 | vPCR |
| UW12712_isTn_us | - CATGGACAAATTAGGACTT - | 55 | vPCR |
| UW12712_isTn_ds | - CAATCGCTACTATAACGCAAAT - | 55 | vPCR |
| cfr_us_BamHI_fw | -TACGGATCCCTTATATTGACAAGAAAGAGGTG- | 54-64.5 | cloning of *cfr(B)* |
| cfr_ds_SalI_rv3 | -TTACGTCGACACTTGAGTGATGCCTATTTTTCT- | 54-64.5 | cloning of *cfr(B)* |
| cfr_UW10882_qPCR_fw | -AGCTGGTTGGGAGTCATTT- | 60 | qPCR |
| cfr_UW10882_qPCR_rv | -TCGCTAATGCTTCTCCCAT- | 60 | qPCR |

Abbreviations: vPCR, verification PCR; qPCR, quantitative PCR
