# Supplementary material for: Detection of a cfr(B) Variant in German Enterococcus faecium Clinical Isolates and the Impact on Linezolid Resistance in Enterococcus spp
Source: PLoS One. 2016 Nov 28;11(11):e0167042. doi: 10.1371/journal.pone.0167042 (PMC5125667; doi:10.1371/journal.pone.0167042)
Supplement: S2 Table — (DOCX) [file pone.0167042.s002.docx]

# S2 Table. MIC determination of *E. faecium* and *E. faecalis* transformed with either *cfr(B)* variants from *E. faecium* or *C. difficile*.

| **Strain** | **MIC^a^ (mg/L)** | | | | | | | | | | | | | | | | | | |
| --- | --- | --- | --- | --- | --- | --- | --- | --- | --- | --- | --- | --- | --- | --- | --- | --- | --- | --- | --- |
|  | **PEN** | **AMP** | **GEN** | **STR** | **VAN** | **TPL** | **DAP** | **CLI** | **ERY** | **CIP** | **MFL** | **SXT** | **TET** | **TGC** | **RIF** | **LZD** | **MUP** | **CMP** | **Q/D** |
| *E. faecium* 64/3 (pEF-25) | 4 | ≤2 | 8 | 32 | ≤1 | ≤1 | ≤0,5 | >8 | >8 | 2 | 1 | ≤0,5 | 1 | ≤0,063 | >4 | 4 | ≤2 | 16 | 2 |
| *E. faecium* 64/3 (pWJB001) | 4 | ≤2 | 8 | 32 | ≤1 | ≤1 | ≤0,5 | >8 | >8 | 2 | 1 | ≤0,5 | 1 | ≤0,063 | >4 | 4 | ≤2 | 16 | 1 |
| *E. faecium* 64/3 (pWJB002) | 4 | ≤2 | 8 | 32 | ≤1 | ≤1 | ≤0,5 | >8 | >8 | 2 | 1 | ≤0,5 | 1 | ≤0,063 | >4 | 4 | ≤2 | 16 | 1 |
| *E. faecalis* JH2-2 (pEF-25) | 4 | ≤2 | 8 | 32 | ≤1 | ≤1 | 1 | >8 | >8 | 1 | 0,25 | 1 | ≤0,5 | ≤0,063 | >4 | ≤2 | >32 | 16 | 32 |
| *E. faecalis* JH2-2 (pWJB001) | 4 | ≤2 | 8 | 32 | ≤1 | ≤1 | 1 | >8 | >8 | 1 | 0,25 | 1 | ≤0,5 | ≤0,063 | >4 | ≤2 | >32 | 16 | 32 |
| *E. faecalis* JH2-2 (pWJB002) | 4 | ≤2 | 8 | 32 | ≤1 | ≤1 | 1 | >8 | >8 | 1 | 0,25 | 1 | ≤0,5 | ≤0,063 | >4 | ≤2 | >32 | 16 | 32 |

^a^MIC determined by broth microdilution; PEN, penicillin; AMP, ampicillin; GEN, gentamicin; STR, streptomycin; VAN, vancomycin; TPL, teicoplanin; DAP, daptomycin; CLI, clindamycin; ERY, erythromycin; CIP, ciprofloxacin; MFL, ; SXT, ; TET, tetracycline; TGC, tigecycline; RIF, rifampicin; LZD, linezolid; MUP, mupirocin; CMP, chloramphenicol; Q/D, quinupristin/dalfopristin.
